# Treatment Failure in Patient with Severe Mpox and Untreated HIV, Maryland, USA

**DOI:** 10.3201/eid2906.230059

**Published:** 2023-06

**Authors:** Evgenii Filippov, Sanchit Duhan, Laura Lehman, Bijeta Keisham, Vishal Sethi

**Affiliations:** Sinai Hospital of Baltimore, Baltimore, Maryland, USA

**Keywords:** mpox, monkeypox virus, HIV/AIDS and other retroviruses, disease outbreaks, viruses, sexually transmitted infections, Baltimore, Maryland, United States

## Abstract

A 33-year-old man in Baltimore, Maryland, USA, with untreated HIV infection had a 74-day course of mpox with multiorgan system involvement and unique clinical findings. In this clinical experience combining 3 novel therapeutic regimens, this patient died from severe mpox in the context of untreated HIV and advanced immunodeficiency.

In July 2022, the World Health Organization declared human mpox an international public health emergency (*1*). Mpox usually has a self-limiting illness course of 2–4 weeks; characteristic rash is the most common symptom and is associated with fever, lymphadenopathy, and fatigue in ≈50% of cases (*2*). Although ≈5.8% of confirmed cases require hospitalization, patients with advanced AIDS might be especially prone to severe mpox and death (*3*).

No medications have a proven benefit for mpox, and experience with available regimens is limited. Four medications are available under clinical trials or the US Food and Drug Administration expanded access protocol (*4*). Two of them, brincidofovir and tecovirimat (TPOXX or ST-246), have shown effectiveness against orthopoxvirus in animal models (*5*,*6*). Both medications have a safe side effect profile in humans (*7*,*8*). According to the Centers for Disease Control and Prevention (CDC), tecovirimat is the first choice and should be taken with fatty meals. For patients who experience clinically significant disease progression while receiving tecovirimat or who experience recrudescence, brincidofovir can be used as an adjunctive therapy. Another medication, cidofovir, can be used in cases of severe monkeypox virus infection, although it has a less favorable safety profile. Vaccinia immune globulin intravenous (VIG-IV) can also be used in severe illness and prophylactically in patients with T-cell deficiency who cannot receive live mpox vaccines (*4*).

We report a case of disseminated mpox treated with those novel drugs in a patient with untreated HIV who was admitted multiple times to different hospitals. Information regarding outside admissions was obtained from electronic medical records.

## The Study

A 33-year-old man with untreated HIV infection sought care at an outside hospital for generalized rash, diarrhea, and odynophagia. His first skin lesion (day 0) appeared 4 days before the hospitalization, and his last sexual contact occurred with a male partner 10 days before hospitalization. He had 25–30 circular, raised lesions (vesicles, pustules, and scabs) measuring 1 × 1 cm distributed over his entire body. Some of the lesions had purulence around the circumference with a dark center. His CD4 count was 25 cells/mm^3^ (reference range 500–1,500 cells/mm^3^) and viral load 678,000 copies/mL (ideal range in HIV infection <20 copies/mL). 

Results of blood cultures at admission were negative. Given the high clinical suspicion of mpox, superficial swab specimens were collected from skin lesions. The viral PCR result was positive for nonvariola orthopoxvirus DNA of mpox on day 6. He was treated with oral tecovirimat (600 mg 2×/d). On day 10, he was discharged with the remainder of the 14-day course and close outpatient follow-up.

Two days later, the patient was readmitted to a different outside hospital for worsening fever and painful skin lesions. He had widespread annular and pustular lesions on the face, scalp, trunk, and extremities. He could not tolerate oral tecovirimat and received further doses intravenously (days 18–23 after onset of lesions). Most of his lesions crusted during this treatment, and the fever subsided. The patient received a 17-day course of tecovirimat before leaving against medical advice on day 23. He was noted to be unaccepting of his HIV status and declined antiretroviral therapy (ART) during each hospital admission.

On day 44, he was readmitted to Sinai Hospital of Baltimore (Baltimore, Maryland, USA) for severe swelling in his right hand, lethargy, and fever, as well as widespread coalescing, painful, and necrotic lesions (>3 cm diameter). Several lesions were ulcerated and open, and he also had a few moist, small vesicular lesions (0.5 × 0.5 cm). CD4 count was 22 cells/mm^3^, HIV viral load was 229,000 copies/mL, and leukocyte count was 11,700 cells/mm^3^ (reference range 4,500–11,000 cells/µL). Ultrasonography of the right arm showed subcutaneous edema but no abscess. He declined ART throughout this hospitalization and initially deferred mpox medications. We empirically treated suspected bacterial superinfection with vancomycin and cefepime, which were later transitioned to linezolid and meropenem secondary to worsening leukocytosis and fevers. 

**Figure 1:**
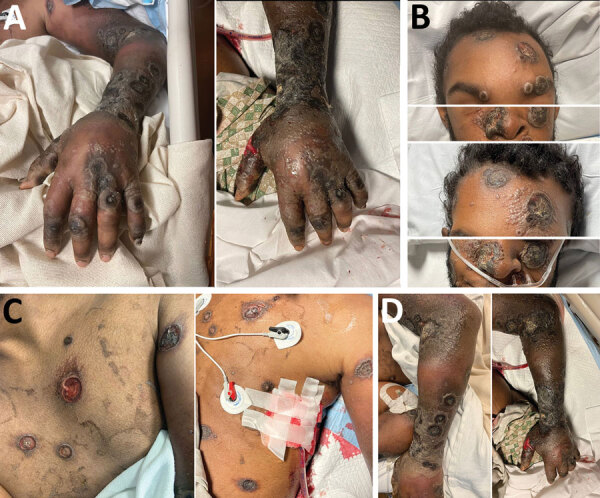
Changes in skin lesions despite aggressive treatment with antiviral medications in patient with severe mpox and untreated HIV, Baltimore, Maryland, USA: A) left hand, B) face, C) trunk, and D) left arm and elbow. Images at left taken on day 65, after 18 days of tecovirimat and 1 dose of brincidofovir. Images at right taken on day 73, after 26 days of tecovirimat, 2 doses of brincidofovir, and 1 dose of vaccinia immune globulin intravenous.

**Figure 2:**
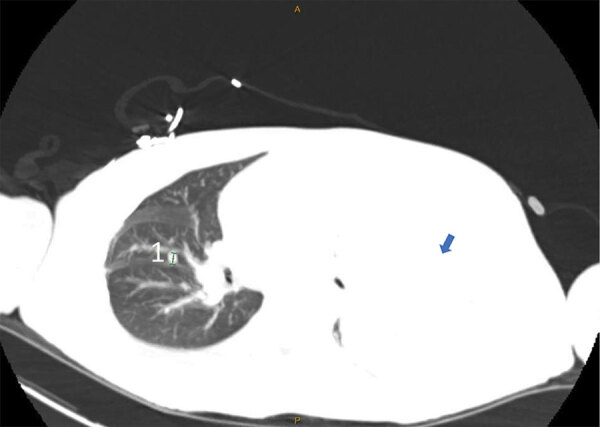
Computed tomography scan of lung showing right-sided pulmonary nodule (marked as 1) and large left pleural effusion (blue arrow) in patient with severe mpox and untreated HIV, Baltimore, Maryland, USA.

**Figure 3:**
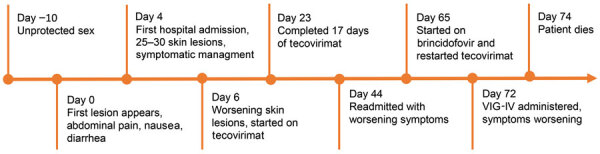
Timeline depicting clinical course of patient with severe mpox and untreated HIV, Baltimore, Maryland, USA. VIG-IV, vaccinia immune globulin intravenous.

By day 60, the patient’s rash involved his left eye and arm. Orbital computed tomography showed soft tissue swelling in anterior globes but no intraorbital abnormalities. On day 65, the patient agreed to begin oral tecovirimat and adjunctive brincidofovir (2 doses given 1 week apart). Still, rash and left arm swelling worsened (Figure 1). On day 72, leukocyte count increased to ≈35,000 cells/µL with a neutrophilic predominance (89%; reference range 43%–70%); liver function tests remained unremarkable, and blood cultures remained negative. He received 1 dose of VIG-IV. The next day, severe respiratory distress developed, and we noted several lung nodules and a large left-sided pleural effusion on imaging (Figure 2). A chest tube was placed. On day 74, the patient died of cardiac arrest. The cause of death was determined to be severe monkeypox virus infection per discussion with CDC and the Maryland health department (Figure 3).

## Conclusions

This case demonstrated severe manifestations of mpox in the setting of untreated HIV with advanced immunodeficiency. The patient had worsening dissemination of skin lesions, presumed superinfection, periorbital swelling, and possible lung involvement. Historically, intratracheal monkeypox virus administration in Macaque monkeys caused rapidly progressive fatal pneumonia (*9*). The pulmonary nodules in this patient closely resemble those seen by Manta et al. (*10*) in a patient with pulmonary mpox. We lack pathologic evidence, but this patient’s lung complications could be secondary to mpox. The role of early imaging and biopsy in identifying those lesions warrants further study.

The body of data regarding the interaction between untreated HIV and mpox is growing. Evidence suggests higher hospitalization rates, severe infections, and increased mortality rates in this subgroup. The current recommendation is to initiate ART at the time mpox is diagnosed (*11*). As seen in this case, treating mpox might not be effective without concurrent ART.

Treatment guidelines for mpox are still developing. An ongoing randomized clinical trial, Study of Tecovirimat for Human Monkeypox Virus, is intended to help determine the efficacy of oral tecovirimat (*12*). Although this patient completed more than the suggested 14-day course of tecovirimat, he intermittently declined several doses, hindering optimal treatment response. Whether the patient was taking tecovirimat with fatty meals at home, as was recommended, was unclear. Brincidofovir was well tolerated without evidence of liver toxicity. The role of initiating intravenous forms of these medications early warrants discussion. Prospective trials with the available agents in various combinations and treatment durations for managing mpox are needed.

Barriers to HIV treatment are still prevalent and include poverty, behavioral health disorders, and substance use disorders. Fear of living with HIV and lack of awareness regarding the benefits of ART also play a role; Dasgupta et al. (*13*) found that 91.5% of patients about whom clinicians sought CDC guidance were persons with HIV not taking ART. Of those, 68% were Black, as was the patient in this study. Only a few states publicly report racial disparities, which could lead to a disproportionate effects of mpox outbreaks, as was seen in the COVID-19 pandemic (*14*,*15*). The specific barriers in our case could not be elucidated.

This case adds to the limited clinical experience with these novel medications in treating mpox. We second CDC recommendations regarding the necessity of ART in patients with HIV and mpox. Barriers to HIV treatment can be detrimental to prognosis and must be preemptively identified and addressed. This case also raises the suspicion of possible pulmonary involvement, which could pave the way for further research into the spread of mpox and systemic involvement. In this case, mpox treatments deployed months into the course of illness, in the setting of untreated HIV, did not appear to alter disease progression to death.
